# Astrocytes: can they be the missing stars linking neuronal activity to neurofunctional imaging signals?

**DOI:** 10.3389/fncel.2013.00021

**Published:** 2013-03-08

**Authors:** Hirac Gurden

**Affiliations:** Imagerie et Modélisation en Neurobiologie et Cancérologie, UMR8165, CNRS, Université Paris Diderot et Paris SudOrsay, France

Functional neuroimaging techniques are currently used in fundamental neuroscience as well as in cognitive neuroscience and clinics to study brain function at large spatial scales. They include Blood Oxygenation Level Dependent-functional Magnetic Resonance Imaging (BOLD-fMRI, Kim and Ogawa, 2012), the gold standard of functional neuroimaging techniques, and many optical techniques including functional Near InfraRed Spectroscopy (fNIRS) in human research or intrinsic/spectroscopic optical signal (IOS) imaging in animal research (Devor et al., 2012). These techniques rely on vascular signals [cerebral blood flow (CBF) and volume and metabolic rate of oxygen consumption] that constitute a proxy for neuronal activity because of the existence of functional hyperemia, a mechanism defined as the matching of vascular changes to the activity level in a given brain area (Iadecola and Nedergaard, 2007). To date, some crucial open questions remain concerning these functional signals, including: what kind of brain activity are they related to? What are their cellular/molecular sources? Can astrocytes, in addition to roles in translating neuronal into vascular activity, also be capable of generating functional neuroimaging signals on their own?

## The disputed sources of BOLD: correlation with synaptic vs. spiking activity

Are BOLD signals correlated to local synaptic activity and/or to spiking activity? Answering this question will bring us a step forward in the understanding of functional connectivity within BOLD maps. Since Logothetis et al. (2001), who combined electrophysiological recordings with fMRI, BOLD signals were thought to be tightly correlated to Local Field Potentials (an electrophysiological signal related to changes in the input and local activities within a given brain structure) and “less correlated” with the output spiking activity. Viswanathan and Freeman (2007) have further shown that BOLD signals are correlated with synaptic activity. However, recent data by Lee et al. (2010) who combined optogenetics with fMRI have revealed that spiking activity can be a predictor of BOLD as efficiently as LFPs. These discrepancies can be due to the study of different species (primate in Logothetis et al. vs. rodent in Lee et al.), different brain structures (visual vs. motor area), and brain states (awake monkey vs. anesthetized mouse). Alternatively, since astrocytes bridge anatomically and functionally neurons with blood vessels (Iadecola and Nedergaard, 2007), they can be considered as an explanation for the coupling of neuronal activity with vascular signals. Indeed, astrocytic processes ensheath synapses as well as blood vessels. In addition, in response to neuronal activity, astrocytes are capable of gliotransmitting active molecules regulating synaptic, metabolic, and hemodynamic activities (Iadecola and Nedergaard, 2007).

Schulz et al. (2012) have recently evaluated how neuronal and astrocytic activities correlate with the BOLD signal using fiber-optic recordings of calcium dyes in the rat somatosensory cortex. They have recorded slow BOLD signals which are systematically accompanied by slow astrocytic calcium signals. This correlation at long time scales (peak activity >3 s following sensory stimulation onset) fits with the *in vivo* recordings of astrocytic calcium dynamics in anesthetized preparations from the ferret visual cortex (>3 s, Schummers et al., 2008) and the mouse olfactory bulb (>1 s, Petzold et al., 2008). Still, astrocytes are capable of fast calcium activity (peak activity 0.5–1 s following sensory stimulation onset) in the somatosensory cortex of anesthetized (Winship et al., 2007) or awake (Dombeck et al., 2007) mice. This faster time scale is in the range of functional hyperemia and related signals such as IOS.

This piece of literature concerning the cellular sources of BOLD shows that further work is needed to identify the local synaptic vs. the spiking activity as being the best correlate/predictor of BOLD as well as the neuronal and/or astrocytic components of these signals. But one thing is sure: BOLD is arising from the vascular compartment. Thus detailed anatomofunctional studies of the neurovascular unit have been undertaken to explore mechanisms making functional hyperemia possible, in order to find some clues about BOLD sources. These studies have led to many conflicting results and conclusions, especially concerning the glutamate signaling pathways which may contribute to activity-dependent regulation of local CBF (Table 1).

**Table 1:** **Examples of receptor candidates for the feed-forward triggering of CBF increase *in vivo***.

	**Support a role in functional hyperemia**	**Argue against a role in functional hyperemia**
**Molecular candidates**		
Neuronal and astrocytic metabotropic glutamate receptors	Takano et al. (2006, SC)	Calcinaghi et al. (2011, SC)
Neuronal and astrocytic ionic glutamate receptors	Offenhauser et al. (2005, Cb); Chaigneau et al. (2007, OB)	Petzold et al. (2008, OB); Scott and Murphy (2012, SC)

I have selected some significant references in the literature that support or argue against a role for each family of molecules in contributing to activity-dependent functional hyperemia in vivo. Cb, cerebellum; OB, olfactory bulb; SC, somatosensory cortex.

## What molecular keys from astrocytes are needed to start the vascular engine in response to neuronal activity?

Among the candidates responsible for functional hyperemia (Attwell et al., 2010; Petzold and Murthy, 2011) much attention has been focused on astrocytic metabotropic glutamate receptors (especially mGluR5) but the importance of calcium dynamics triggered by these receptors in astrocytic physiology is a matter of some debate (Agulhon et al., 2008; Calcinaghi et al., 2011). Astrocytic glutamate transporters (GluTs) constitute another exciting candidate for the function of the neurovascular unit. GluTs activity is required for: (1) glutamate clearance and regulation of activity at excitatory synapses (2) glucose uptake to build up glycogen stores, synthetize glutamate, and produce lactate (3) glutamine recycling to restore presynaptic glutamate vesicles (Danbolt, 2001). GluTs are glutamate/sodium co-transporters. Intra-astrocytic sodium increases glucose uptake and subsequent transformation into lactate which is released in the extracellular medium. Lactate could serve both as an energetic substrate (Pellerin et al., 2007), a regulator of CBF (Gordon et al., 2008), and also as a mediator of metabolic information for synapses (Bergersen and Gjedde, 2012). Thus GluTs constitute a cross road for the coordinated control of information and energy processing (Martin et al., 2012). Can GluTs lead to functional signals? This question was addressed by two groups who impaired GluTs activity and followed changes in complex IOS signals. IOS were measured as red light absorption changes due to vascular changes in CBF and volume as well as light scattering. In both cases, the authors mapped sensory activity (rat olfactory bulb, Gurden et al., 2006; ferret visual cortex, Schummers et al., 2008) and demonstrated that ionotropic glutamate receptors inhibition did not affect IOS, whereas TBOA, a blocker of GluTs, did. In addition, Schummers et al. showed that astrocytic calcium signals were reduced by TBOA. This molecule also inhibits functional hyperemia in the olfactory bulb (Petzold et al., 2008). How GluTs activity is mechanistically linked to functional hyperemia is an open question. GluT-induced IOS (Gurden et al., 2006) and CBF changes (Petzold et al., 2008) were not mediated indirectly via glutamate receptors nor presynaptic GABA-B receptors. Sodium waves in the astrocytic network are triggered by GluTs (Bernardinelli et al., 2004) and it seems very likely that some interaction with calcium dynamics (Schummers et al., 2008) may be responsible for GluT-induced CBF changes. Interestingly, plasma level changes in lactate and pyruvate levels were found to be reflected in BOLD signals (von Pföstl et al., 2012) but these results will have to be confirmed during the activation of local brain networks. Finally it is not known whether any of the candidates responsible for functional hyperemia and/or for functional optical signals (including GluTs) are indeed related to BOLD signals. For example, no activation or impairment of a specific neurotransmitter system was undertaken during BOLD recordings.

Since astrocytes (1) express receptors for neuromodulators such as noradrenaline, which is known to regulate both the vascular tone (Hamel, 2006; Bekar et al., 2012) and astrocytic activity (Bekar et al., 2008) and (2) have their own specific calcium dynamics, which do not match in space and time the neuronal activity response to visual stimuli (Schummers et al., 2008), can they generate functional signals (Figley and Stroman, 2011)?

## Solving the cellular basis of functional neuroimaging signals: specific neuronal and astrocytic optogenetics coupled to BOLD and optical imaging

To further understand the roles of astrocytes vs. neurons in BOLD signals, the following approaches could be combined: (1) *in vivo* imaging of awake mice to avoid any anesthetic effects on neuronal and astrocytic activity (Thrane, 2012), (2) specific optogenetic targeting and stimulation of either astrocytes or excitatory neurons and interneurons and manipulate the temporal sequence of activation (3) fiber optics to stimulate and monitor activity dynamics in each of these cellular populations during BOLD recordings. All of these approaches exist in the literature separately. For example, Desai et al. (2011) have run experiments on awake mice in fMRI and Dombeck et al. (2007) have performed optical imaging of astrocytic activity in awake mice. Schulz et al. (2012) have recorded simultaneous BOLD and calcium signals in neurons and astrocytes using fiber optics. In addition, optogenetic stimulation of parvalbumin interneurons in the mouse barrel cortex (Kahn et al., 2011) or pyramidal cells in the motor cortex (Lee et al., 2010), with both type of cells expressing Channelrhodopsin-2 (ChR2), has been shown to produce BOLD signals. These studies establish a causal relationship between the activation of defined neuronal populations and BOLD signals. ChR2 optogenetics was also used in astrocytes by Gourine et al. (2010) who studied respiratory regulation in the brainstem. The next step is thus to express ChR2 specifically in cortical astrocytes and stimulate them to test whether they can lead to significant BOLD signals. New tools such as genetically engineered calcium sensors (GCaMPs) and effector proteins (ChRs) expressed in astrocytes (Figueiredo et al., 2011) will also make possible the accurate monitoring and the specific stimulation/inhibition of astrocytic activity during BOLD recordings. For the spatiotemporal control of cellular activation, electrical microstimulation (Tolias et al., 2005) and optogenetic (Lee et al., 2010; Kahn et al., 2011) stimulations were used. In addition to these techniques, DREADD (“Designer Receptor Exclusively Activated by a Designer Drug”) would help to pinpoint specific transmitter systems involved in BOLD generation (Nawaratne et al., 2008). The prototype DREADD is a variant of a human muscarinic receptor that is no longer activated by its endogenous ligand, acetylcholine, but by exogenous clozapine-N-oxide, an inert metabolite of the antipsychotic drug clozapine (Dong et al., 2010). A combined chemical–genetic approach, in which mouse genetics limit the spatial expression of the DREADD to a specific cellular type and the designer drug (intraperitoneal injection) provides temporal control over G protein–coupled receptors activity could help describing the links between activity of a particular receptor and BOLD signals.

In conclusion, determining the relative role of astrocytes vs. neurons in functional hyperemia and BOLD signals is still an exciting challenge. Since technical tools are now available, experiments should follow soon to lighten up the cellular dark side of BOLD.
